# Increased sediment loads cause non-linear decreases in seagrass suitable habitat extent

**DOI:** 10.1371/journal.pone.0187284

**Published:** 2017-11-10

**Authors:** Megan Irene Saunders, Scott Atkinson, Carissa Joy Klein, Tony Weber, Hugh P. Possingham

**Affiliations:** 1 Centre for Biodiversity and Conservation Science, The University of Queensland, St. Lucia, QLD, Australia; 2 Australian Research Council (ARC) Centre of Excellence in Environmental Decisions, The University of Queensland, St Lucia, QLD, Australia; 3 School of Earth and Environmental Sciences, The University of Queensland, St. Lucia, QLD, Australia; 4 School of Chemical Engineering, The University of Queensland, St. Lucia, QLD, Australia; 5 Alluvium Consulting Australia, Fortitude Valley, QLD, Australia; 6 The Nature Conservancy, Arlington, VA, United States of America; University of Sydney, AUSTRALIA

## Abstract

Land-based activities, including deforestation, agriculture, and urbanisation, cause increased erosion, reduced inland and coastal water quality, and subsequent loss or degradation of downstream coastal marine ecosystems. Quantitative approaches to link sediment loads from catchments to metrics of downstream marine ecosystem state are required to calculate the cost effectiveness of taking conservation actions on land to benefits accrued in the ocean. Here we quantify the relationship between sediment loads derived from landscapes to habitat suitability of seagrass meadows in Moreton Bay, Queensland, Australia. We use the following approach: (1) a catchment hydrological model generates sediment loads; (2) a statistical model links sediment loads to water clarity at monthly time-steps; (3) a species distribution model (SDM) factors in water clarity, bathymetry, wave height, and substrate suitability to predict seagrass habitat suitability at monthly time-steps; and (4) a statistical model quantifies the effect of sediment loads on area of seagrass suitable habitat in a given year. The relationship between sediment loads and seagrass suitable habitat is non-linear: large increases in sediment have a disproportionately large negative impact on availability of seagrass suitable habitat. Varying the temporal scale of analysis (monthly vs. yearly), or varying the threshold value used to delineate predicted seagrass presence vs. absence, both affect the magnitude, but not the overall shape, of the relationship between sediment loads and seagrass suitable habitat area. Quantifying the link between sediment produced from catchments and extent of downstream marine ecosystems allows assessment of the relative costs and benefits of taking conservation actions on land or in the ocean, respectively, to marine ecosystems.

## Introduction

Marine ecosystems worldwide are under threat from a range of human activities occurring both in the ocean and on land [1]. As human populations continue to grow, particularly near coastlines, coastal marine ecosystems are increasingly exposed to multiple threats [2]. The conservation of coastal ecosystems has primarily focused on mitigating marine-based threats, such as overfishing, through implementation of marine protected areas and/or regulating fisheries [3]. However, land-based activities, such as agriculture, mining, and road construction, have a substantial impact on coastal marine ecosystems due to increased supply of sediments, nutrients, and other pollutants to oceans [1, 4–9]. In many locations, land-based activities are considered to be among the most significant threats to marine ecosystems (e.g. Great Barrier Reef World Heritage Area [10]). Thus, effective management of coastal marine ecosystems requires consideration of both land- and sea-based threats [3, 11–17].

As with any conservation problem, budget constraints mean that we must prioritise what and where we invest. To accommodate the growing need to consider threats from both the land and sea, conservation planners have developed a range of new approaches to help prioritise investment for marine ecosystems [11]. In general, there are two types of approaches: (1) Threat-based, where the objective is to maximise reduction of threats (e.g., sediment run-off) [3, 9, 18]; and (2) Outcome-based, where the objective is to maintain or improve the state of a species or ecosystem through the reduction of threats [12, 14]. Outcome-based approaches are preferable because they directly relate threats to ecological metrics, but significant challenges have prevented their development and use in marine conservation planning. Data and models are required to quantify the links between: (1) the reduction in run-off required to reduce threats on receiving marine environments, and (2) the amount of change in the marine ecosystem triggered by such reduction. Otherwise stated, we need models that can predict how much particular metrics of the marine environment will improve based on conservation actions taken on land.

Regardless of approach, it is necessary to predict the amount and type of run-off produced by a catchment, and a range of models have been developed which support this step in the planning phase, including the Invest and Source modelling suites [15, 19–22]. To address an outcome-based objective, two steps are required: (1) Predict generation and dispersal of run-off, and; (2) Predict the impact of run-off on the relevant marine ecosystem. Most land-sea planning approaches rely on a linear decay model to estimate the spatial impact of run-off plumes, following methods initially developed in Halpern et al. [1], and subsequently applied in other studies [9, 14, 15]. Recent studies have implemented more complex plume models that account for currents and bathymetry [23, 24]. The next step–estimating how run-off affects the state of marine ecosystems–is typically neglected. A few studies have attempted to model the relative impacts of run-off and fishing on coral reef ecosystems, but make significant assumptions [14, 23]. Others have applied a risk framework to relate exposure to sediment plumes to percent cover of seagrass and coral [25]. Here, we progress this area of research by developing a model of the influence of sediment loads on habitat suitability of subtropical seagrass meadows, which is applicable to other benthic photosynthetic organisms, such as coral or algae.

Seagrass meadows provide a wealth of ecosystem functions and services, yet are degrading rapidly due to multiple threats [26, 27]. Seagrasses provide habitat for endangered species such as marine turtles and dugongs, as well as species supporting commercial and recreational fisheries [28, 29]. By reducing wave energy and trapping particulate matter, seagrasses protect and stabilise shorelines [30], and sequester carbon from the atmosphere, storing it in the seafloor, thus helping to mitigate climate change [31, 32]. Changes in land-use have caused increased sediment erosion and eutrophication of near-shore waters, which has led to extensive degradation and loss of seagrass meadows [27, 33]. Suspended sediments reduce light penetration in water, which decreases depth range, extent, and shoot density, and alters species composition, of seagrass [34–37].

The objective of this study was to quantify the effect of sediment loads on the extent of seagrass suitable habitat in a coastal embayment. A suite of modelling tools informed by spatial and time-series data were adopted and developed for this purpose. The modelling approach and output can provide a basis for understanding how changes in sediment loads, due to either variability in rainfall or modifications to land-uses in catchments, may affect coastal ecosystems. We use the model successes and limitations to highlight areas for future research.

## Materials and methods

### Study system

**Fig 1:**
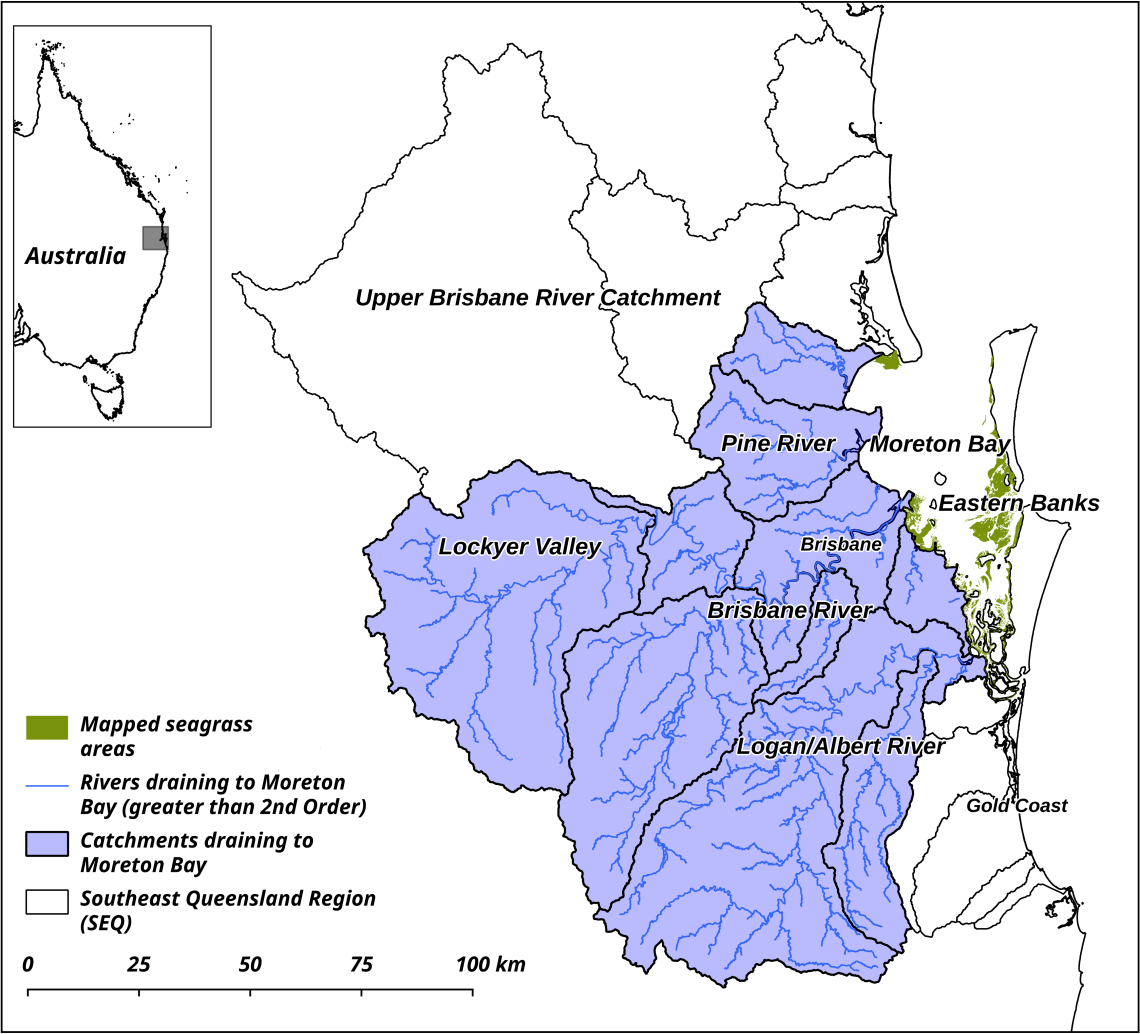
Study site. Study site in Moreton Bay and surrounding catchments in Queensland, Australia. Seagrass data were obtained from [42, 43], and all other data are courtesy of the Government of Queensland.

The study was conducted in Moreton Bay and surrounding catchments, in Queensland, Australia (Lat: -27.0–28.3; Lon: 151.9–153.4) (Fig 1). Moreton Bay is a shallow semi-enclosed embayment adjacent to Brisbane, the capital city of Queensland (population ca. 2.3m). The climate is subtropical, with hot and humid Austral summers and cool dry winters [38]. Six rivers drain from the adjacent catchments, which have been extensively modified over the previous 150 years for agriculture and development [39]. Sediment run-off creates turbid conditions, particularly inshore [38]. Sediment loads from the catchments are 280,000 T per year in typical years [40]. Extensive wetlands and mudflats form habitat and feeding grounds for vulnerable and threatened species including green turtles (*Chelonia mydas*), dugongs (*Dugong dugon*), and migratory shorebirds [38]. Over 200 km^2^ of seagrass meadows, comprised of seven species, have been well described from satellite imagery and field surveys [41, 42].

### Model overview

**Fig 2:**
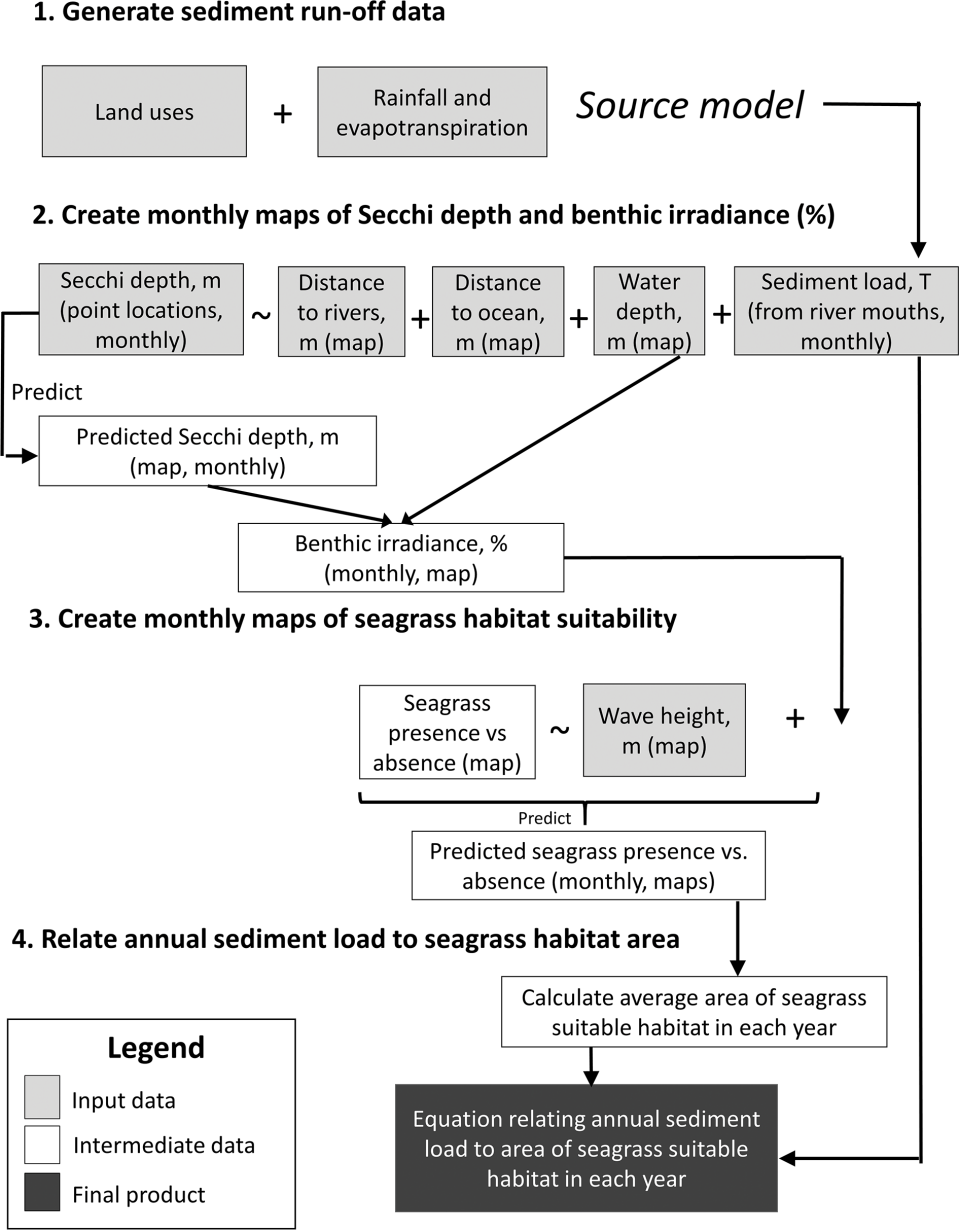
Work flow diagram. Work flow diagram for model of impacts of sediment loads on seagrass habitat suitability in Moreton Bay, Queensland, Australia.

The modelling approach was based on species distribution modelling (SDM) methods developed in Saunders et al. [44] and subsequently adopted in later studies [45, 46]. In [44], a map of coastal water clarity is derived from point measurements of Secchi depth and rasterised data sets of water depth, distance to open ocean (assumed to be a proxy for clear water), and distance to river mouths (i.e., sources of turbid water). The map of water clarity is then used to predict benthic light availability, which, in combination with a map of significant wave height, is used to predict habitat suitability for seagrass. Here, we expand this approach substantially to include temporal variability in sediment loads—which influence water clarity—to estimate temporal variability in seagrass suitable habitat area (Fig 2) as follows. (1) A catchment model [47] generates time-series data of sediment run-off; (2) A statistical model, expanded from [44], relates observed water clarity to sediment loads, water depth, and distance to sources of clear and turbid water, and is used to build time-series maps of water clarity and benthic irradiance; (3) A statistical model, developed in [44], calculates the probability of seagrass presence vs. absence as a function of % surface light availability and wave height and is used to generate time-series of seagrass suitable habitat maps; (4) A statistical model, developed for the present study, estimates the effect of sediment loads on seagrass suitable habitat (Fig 2). Each grid cell represents 100 x 100 m. Analyses were conducted using the Source model, R version 3.2.2, and R Studio Version 1.0.143. Detailed information on the input data and each step in the modelling processes is provided below.

### Input data

**Fig 3:**
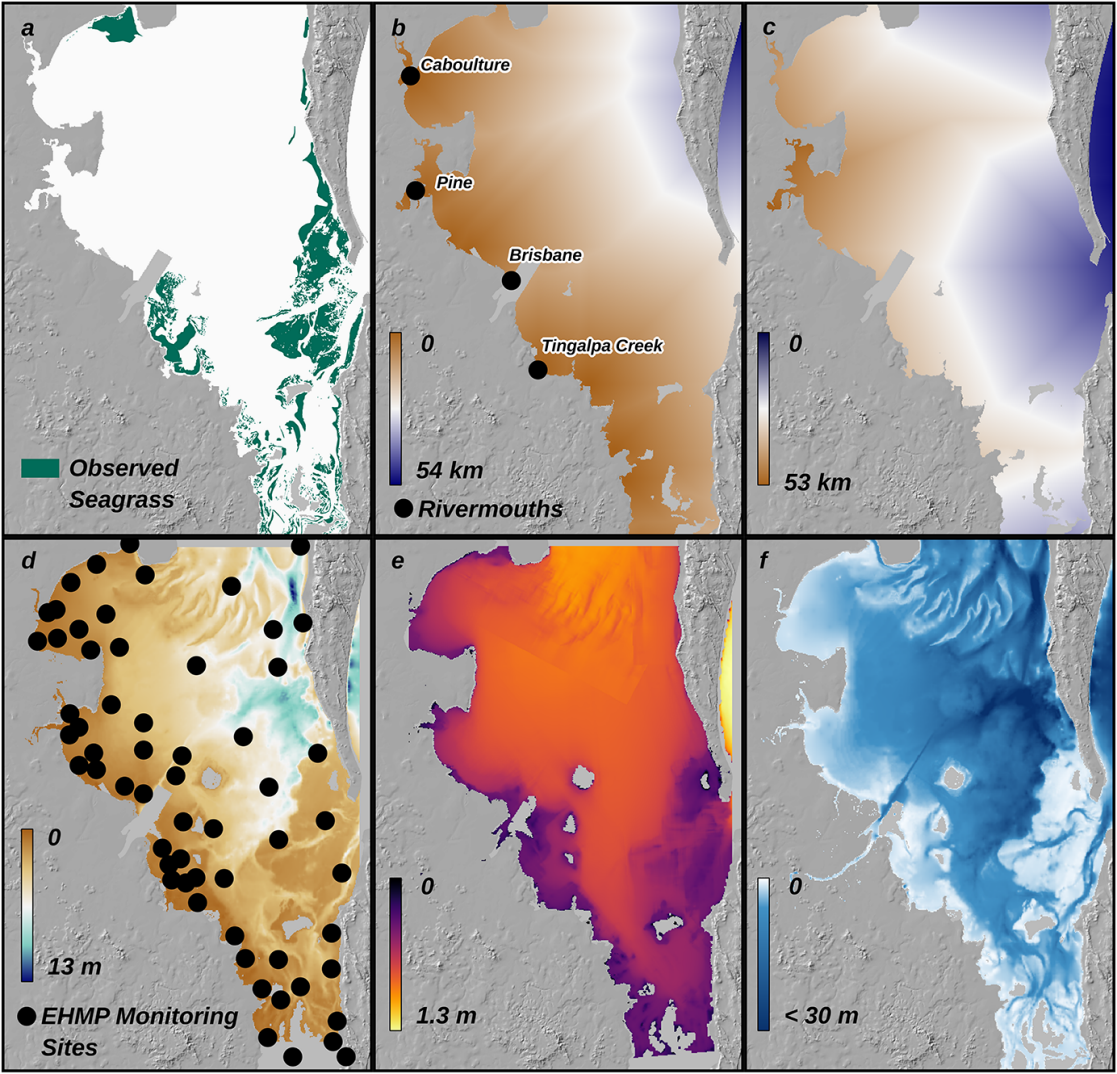
Input data. Data used to model habitat suitability for seagrass in Moreton Bay, Australia: (a) observed seagrass extent in 2004 [42]; (b) distance to rivermouths; (c) distance to open ocean; (d) water quality monitoring sites (dots) and map of modelled Secchi Depth for July 2004; (e) modelled upper 90^th^ percentile of significant wave heights [46, 59]; (f) water depth [63].

Gridded daily rainfall and potential evapotranspiration at 5 x 5 km resolution were obtained from the Scientific Information for Land Owners (SILO) database [48]. Data on land uses (1:50,000) were obtained from the Queensland Land Use Mapping Project [49]. Data for seagrass presence vs. absence at 30 m resolution were obtained from Roelfsema et al. [42, 50] (Fig 3A). The modelling approach that we adapted for the present study assumes that the habitat data are binomially distributed; therefore, we scored the seagrass data as 0 for absent and 1 for present, with seagrass considered as 'present' if seagrass cover was greater than 0% [44]. Our model is therefore trained to predict the areas where seagrass of any species, biomass or cover level, could theoretically occur based on environmental conditions. Developing predictive models of the impact of sediment loads on seagrass which incorporate metrics other than presence vs. absence, such as species [51], cover [52], or biomass [53], are beyond the scope of this study, but are an important area of future research. Water clarity was assumed to be influenced by the distance to open ocean (source of clear water) and river mouths (source of turbid water), as they are likely to influence water clarity [54, 55]. The locations of river mouths, defined as ≥4^th^ order streams [56, 57], flowing into Moreton Bay were identified using the Queensland Waterways for Waterworks dataset [58] (Fig 3B). Open ocean was defined by the 30m depth contour (see *bathymetry*, below) (Fig 3C). Path-distance matrices, which account for barriers to water movement, were used to determine the least-cost (shortest) Euclidean distance at each location to a river mouth and open ocean, respectively, using the Cost Distance Matrix tool in ArcMap. This method accounts for barriers to water movement imposed by shorelines. Water quality data were obtained from the Monitoring Program at Healthy Land and Water (http://hlw.org.au/report-card/monitoring-program) for February 2000 to July 2013 (Fig 3D). Data were sampled at 51 sites in 2000, increasing to 91 sites in 2013. Spatial data layers for Secchi depth were generated based on these data as outlined below. A synoptic map of significant wave height (*H*_*s*_) (Fig 3E) was obtained from Callaghan et al. [46, 59]. This dataset was produced using the Simulating WAves Nearshore (SWAN) wave generation and propagation model [60, 61]. Data represent the upper 90^th^ percentile of wave heights over a 10 year period; this metric was the best predictor of seagrass presence vs. absence in our previous modelling in Moreton Bay and on Lizard Island, Great Barrier Reef [44, 62]. This likely reflects the relative importance of larger waves on limiting habitat suitability for seagrass. Data for water depth at 100 m resolution were obtained from 3D-GBR [63] (Fig 3F). Sources, resolution, limitations and causes of variability in input data are summarised in Table 1.

**Table 1:** Data sources, resolution and uncertainty.

Data	Source	Spatial resolution	Cause of Uncertainty	Uncertainty
Rainfall & evapotranspiration	SILO database [48]	5 km	Spatial interpolation	Annual RMSE 1–34% [64]
Land uses	Queensland Land Use Mapping Project [65]	1:50,000	Accuracy of classification procedure	Total accuracy 0.89–0.95%
Seagrass maps	Roelfsema et al. [42, 50]	30 m	Accuracy of classification procedure	> 75% of Moreton Bay was mapped with high categorical reliability [42]
Distance to river mouths and open ocean	This study; based on Queensland Waterways for Waterway barrier works dataset [58]	100 m	Assumption that 30 m contour represents the location of clear water sources and that sediment plume dispersal is spatially even	Features in the waterways dataset are generally accurate, with some fine scale variability from observed values. Unknown uncertainty in the distance to rivers or ocean layers.
Secchi depth	Healthy Waterways Ecosystems Health Monitoring Program	51–91 points—used to predict at 100 m resolution	Averaging over 6 months for initial model building; spatial interpolation for monthly maps	>80% of spatial variability [44] and 57% of temporal and spatial variability (This study) explained by models
Significant wave height	SWAN Model [44, 46, 59]	100 m	Averaging over time; errors in bathymetric data; assumptions about physical process	84% of variability in observed wave height explained by model
Bathymetry	3D GBR [63]	100 m	Interpolation amongst contour lines	Vertical uncertainty = ± 0.5 m
Sediment loads	This study	3 points for primary rivers	Assumptions about physical processes	Typically less than +/-20% of measured loads

Data sources, resolution and uncertainty used to calibrate and validate models linking sediment loads to seagrass suitable habitat extent in Moreton Bay, Queensland, Australia.

### Modelling approach

#### Generation of sediment load data (Step 1)

Terrestrial sediment input into Moreton Bay was estimated using modelled daily sediment discharge data from the Source model for the catchments of Southeast Queensland. The Source modelling framework [47] simulates catchment characteristics and hydrological responses to rainfall, and evaluates the impacts of land use change and management practices on pollutant loads. Calibration and verification is conducted to ensure that the numerical model adequately represents the study area. The model is forced by rainfall and potential evapotranspiration based on land-uses. Pollutant characteristics are represented largely as event mean concentrations and baseflow concentrations parameterised through event monitoring data for the region. The translation of climate, land use and pollutant concentrations into runoff and pollutant load is completed on a daily timestep through the application of the SIMHYD rainfall runoff model [21]. The model of Southeast Queensland has been developed in the Source modelling framework to build on catchment research, monitoring and modelling that has been undertaken in the region over the last 20 years. Calibration of the model and comparison with other analysis methods [66] shows that the developed model is robust in predicting sediment loads to receiving waters of the region. While other methods and models are available, the Source model of the Southeast Queensland catchments has been regularly tested for suitability in answering key modelling questions for the region and is being continuously updated. Data exported from the model included total suspended solids (TSS, in Tonnes, hereafter ‘sediment’) outputs for the Lower Brisbane, Pine, and Logan/Albert rivers, for January 1980 to June 2014. Sediment data were summed across the three rivers, and aggregated to monthly values by summing dates within a month to match the temporal resolution of the water clarity data.

#### Modelling impact of sediment loads on water clarity and benthic light availability (Step 2)

Statistical model of Secchi depth: Secchi depth (*Z*_*SD*_) was modelled by fitting a Linear Model to Secchi depth, with sediment (*S*), depth (*z*), distance to rivers (*D*_*R*_), and distance to open ocean (*D*_*O*_) as predictor variables. This model was adapted from [44], where a predictive model of *Z*_*SD*_ was developed, and which took the same form but did not include *S*. Data for *S* were comprised of one value for all locations in each month; all other data varied among grid cells, but were consistent across time. Interactions between predictor variables were not accounted for due to the high spatial correlation between the parameters.

Spatial layers for Secchi depth and benthic irradiance: Coefficients from the statistical model of Secchi depth were used to predict *Z*_*SD*_ at all other locations for each month from February 2000 to March 2013 based on sediment in each month and the maps of depth, distance to rivers and distance to ocean.

Benthic light availability: Spatial layers of benthic light availability for each month were created using the data layers for Secchi depth and water depth. Irradiance *I* (W m^2^ s^-1^) at depth *z* of a given location depends upon water clarity and depth (m), and is described by the Lambert-Beer equation [67]:

$$I(Z)=I_{0}e^{-k_{d}Z}$$ (1)

where *I*_*0*_ is surface irradiance (W m^2^ s^-1^) and *K*_*d*_ is the diffuse attenuation coefficient (m^-1^) which represents water clarity. Percent light available at water depth *z* can be estimated by *I(z)*/*I*_*0*_. *K*_*d*_ is a function of Secchi depth (*Z*_*SD*_) according to:

$$K_{d}=\frac{\lambda }{Z_{SD}}$$ (2)

where *λ* is approximately 1.7 [68].

#### Habitat distribution model (Step 3)

Model development: Seagrass habitat distribution modelling methods were developed based on Saunders et al. [44] and expanded to include the influence of temporal variability in sediment loads. The presence or absence of seagrass (mapped in July 2004, [42]) was predicted by fitting a Generalised Linear Model (GLM) assuming a binomial distribution to the input data, with the logarithm of average percent surface irradiance in the 6 months prior to seagrass mapping (‘light’, described above), significant wave height (‘waves’) [46, 59], and the interaction between light and waves, as predictor variables. A spatial autocorrelation term was not included because the purpose of the model was to predict past (or future) conditions where the assumptions of the spatial autocorrelation term would have been violated. If spatial autocorrelation had been factored in the results would have been more conservative; e.g., predicted seagrass habitat area would vary less over time.

Model validation: Model performance and accuracy were assessed using the R package 'PresenceAbsence' [69]. A threshold cut-off value, where probability values above the threshold are considered present, and below the threshold are considered absent, was calculated to be 0.22. This was selected by maximising ‘kappa’, a measure of correctly classifying presence or absence while accounting for chance agreements. A confusion matrix comparing all observed and predicted seagrass presence vs. absence data based on that threshold was generated to assess model performance.

Model implementation: The habitat distribution model was used to predict the probability of a grid cell containing seagrass suitable habitat for each month from February 2000 to March 2013. In each grid cell the probability of seagrass occurring (*p*_*i*_) was determined by applying the logit transformation [70]:

$$p_{i}=\frac{e^{g(x_{1})}}{1+e^{g(x_{i})}}$$ (3)

where *g(x*_*i*_*)* is the linear predictor fitted by the logistic regression. Coefficients of the model were estimated using seagrass and sediment data from 2004. The fitted model was then used to predict probability of seagrass presence in other time periods.

#### Relationship between annual sediment loads and predicted seagrass suitable habitat area (Step 4)

The ultimate aim of the model was to determine the relationship between the tonnes of sediment delivered to the ocean and the area of seagrass habitat supported. To do so, the area of seagrass suitable habitat in each month was calculated by summing the number of 1 Hectare cells where seagrass was predicted to occur for each month; monthly values were then averaged over the wet season of each year (Nov to March) as an estimate of the habitat suitability over the year running from July to June. Lower water clarity in the wet season causes seagrass meadows to contract, with recovery occurring once conditions improve if seed banks are present [71]. Therefore, in southeast Queensland, the conditions most limiting for seagrass are in the wet summer season, when the majority of sediment is delivered to the ocean. The area of seagrass suitable habitat identified in each year was plotted as a function of the tonnes of sediment in each year. Annual sediment load for this application was estimated as 24% more than the sediment load in the wet season, because 76% of the sediment discharged to ocean in a given year at our study site occurs during the wet season, which occurs during the Austral summer (see Results). The effect of sediment load on seagrass suitable habitat area was estimated using a linear model, with habitat area as the response variable and annual sediment and/or sediment^2^ as the predictor variable(s). A model simplification procedure was conducted to select the model which minimized BIC.

### Sensitivity analysis

We tested the sensitivity of the model performance to (1) the temporal scale (monthly, annual) of the analyses in Step 4, and (2) the selection of the threshold value used in the habitat distribution model in Step 3. We tested the sensitivity of the model output to the effect of temporal scale by conducting Step 4 for data plotted over annual time-scales, as described above, as well as monthly time-scales. To test for the effect of the threshold value we repeated Materials and Methods Step 3, using each of four different threshold values for probability of occurrence of seagrass which were selected using the ‘optimal.thresholds’ function in the ‘PresenceAbsence’ package in R [69]. This technique optimises model performance based on up to 10 different criteria. For the ‘main’ results we optimised model performance based on kappa, because it is one of the most commonly used metrics to assess model performance in SDMs [72]. For the sensitivity analysis, we tested the model performance using three other criteria for selecting the threshold value: maximising the percent correctly classified (MaxPC); comparing model sensitivity to specificity (SensSpec); and a default value which would be used if there were other information available. For the present study the threshold values that were selected based on each metric were: Kappa = 0.22; MaxPC = 0.44; Sens = Spec = 0.11; and Default = 0.5. Other metrics can be used to predict the optimal threshold value, but for our study these four spanned the range of values selected using any criteria (0.11–0.5). Using each of the four threshold values independently we completed the modelling process in Steps 3 and 4 for both the monthly and the yearly data sets.

## Results

### Sediment loads

**Fig 4:**
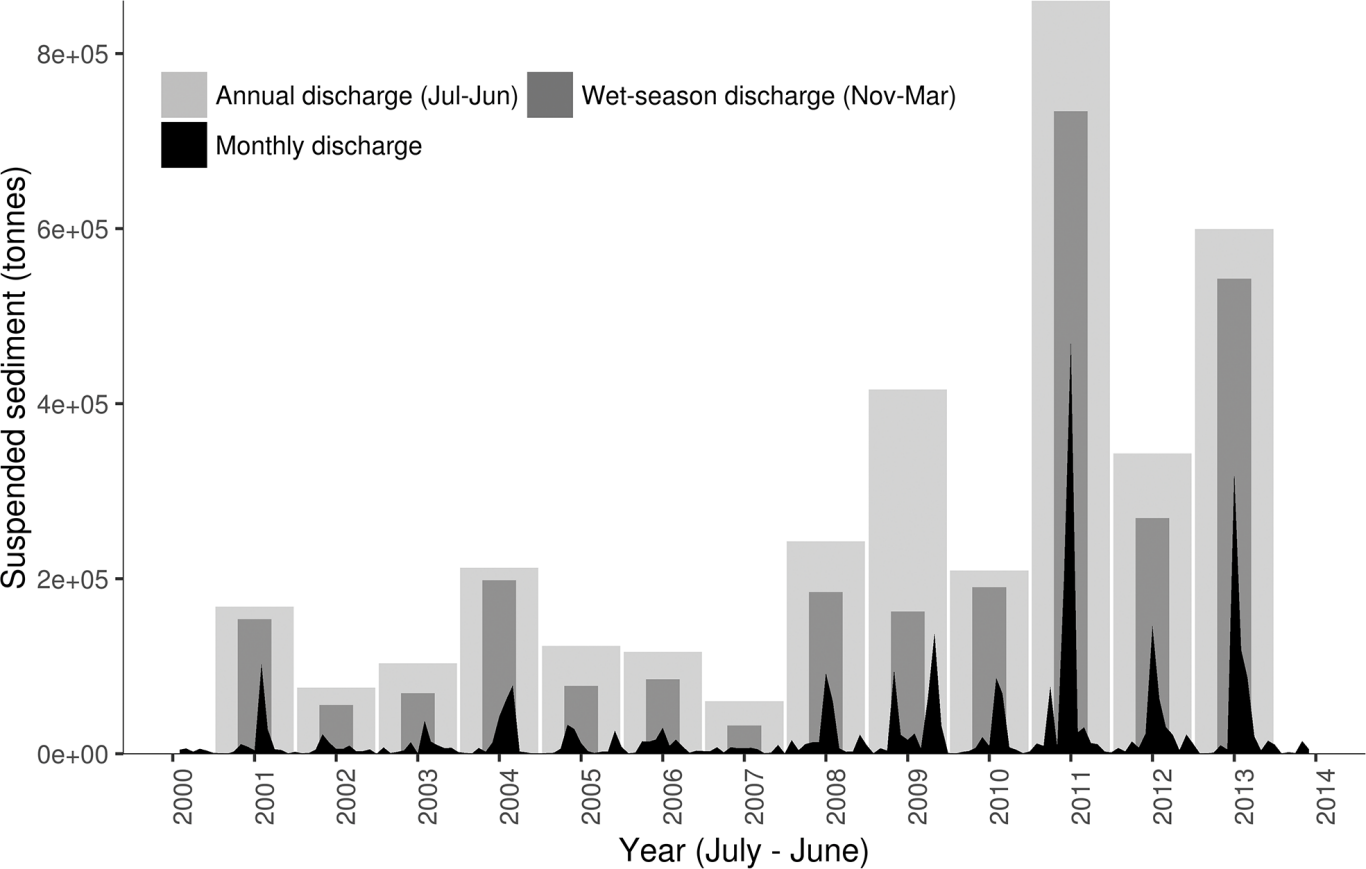
Sediment loads. Modelled suspended sediments (tonnes) delivered to Moreton Bay, Queensland, Australia.

Modelled annual sediment loads varied from 60,000 T yr^-1^ in 2007 to 860,000 T yr^-1^ in 2011 (Fig 4) (S1 Table). There was large interannual variability in sediment discharge, with relatively low sediment discharge in 2000–2010, increasing in later years. Heavy rainfall caused flood conditions in January 2011 [73, 74], resulting in a several fold increase in the amount of sediment eroded from channels and delivered to floodplains [74] and the ocean in that year. On average 76% of the sediment discharge occurred during the austral wet seasons from November to March (Fig 4).

### Water clarity and benthic light availability

Sediment (*S*), distance to rivers (*D*_*R*_), distance to ocean (*D*_*O*_), and water depth (*z*) explained 57% of the variability in Secchi depth (*Z*_*SD*_) (Adjusted R^2^ = 0.57, p < 0.001). The fitted model was:

Z^SD=0.842−4.56×10−6(S)−0.226(z)−7.32×10−6(DO)+8.30×10−5(DR) (4)

As indicated by the relative magnitude of the model coefficients, the majority of variability in Secchi depth is explained by *z*, followed by *D*_*R*_, *T*_*SS*_, and *D*_*O*_.

### Seagrass distribution model

**Fig 5:**
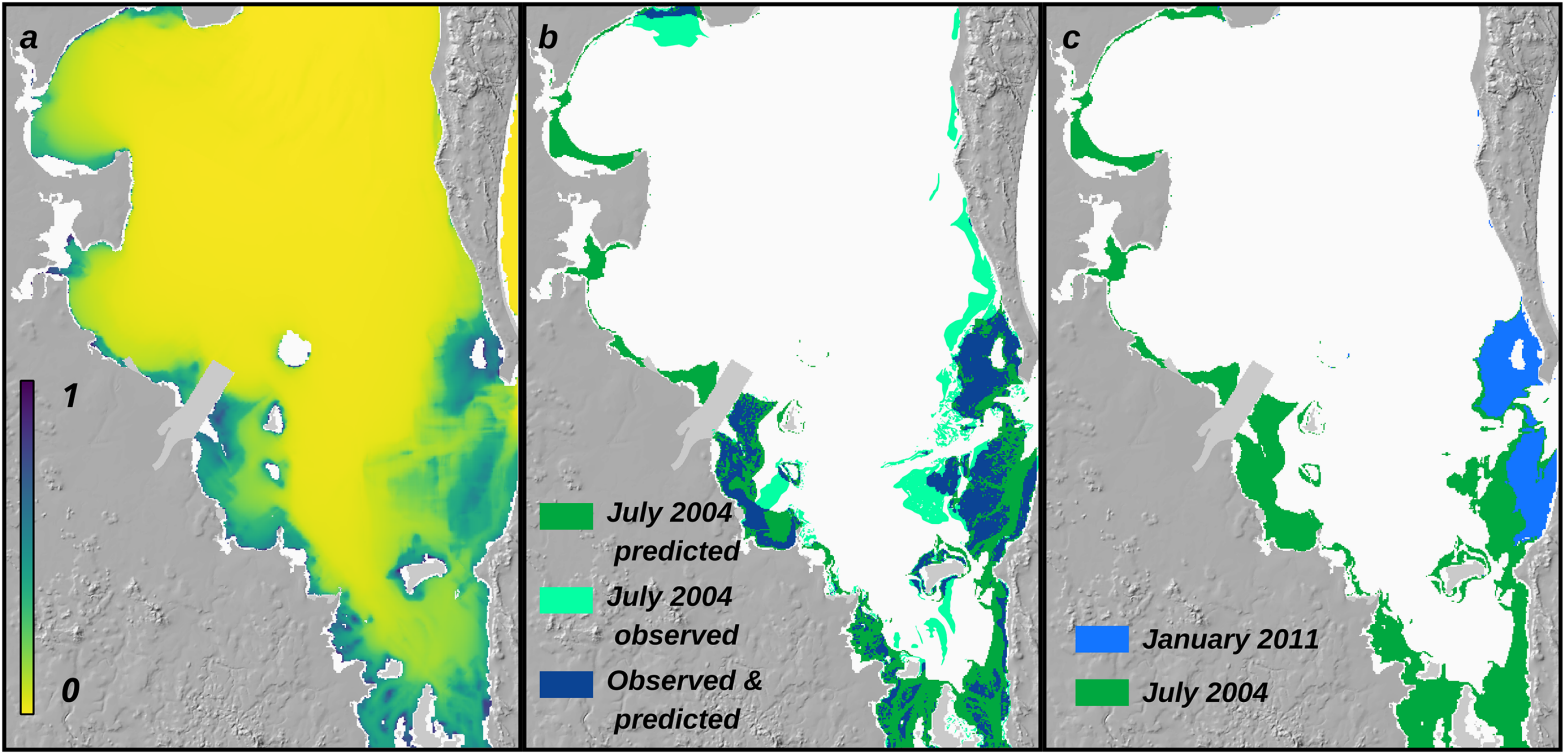
Model output. Output of predictive model of seagrass suitable habitat for Moreton Bay, Queensland, Australia: (a) probability of seagrass presence in a representative year (July 2010); (b) observed and predicted seagrass extent in July 2004; (c) map of predicted seagrass presence in January 2011 (heavy flooding) vs. July 2004 (drought).

Here we present an overview of results of the seagrass distribution model and direct the reader towards the earlier publications for detailed information on model performance [44–46]. In the present application the input data were slightly different, due to small differences in the model domain, and because the Secchi depth layer factored in sediment loads. The linear predictor *g(x*_*i*_*)* of the model [70] took the form:

g(xi)=2.28+0.375(L)−7.89(HS)−0.415(HS×L) (5)

where *z* is depth, *H*_*s*_ is waves, and *L* is light. The model predicts higher probability of seagrass presence in shallow water areas at the coast and in the clear water regions to the east (Fig 5A). Applying the threshold cut-off value for presence vs. absence generates a map of seagrass suitable habitat presence that represents the overall patterns of observed seagrass presence data for 2004 (Fig 5B). Percent correctly classified (PCC) was 85%, indicating that the model correctly estimated seagrass presences and absences in 85% of locations. Errors of commission were higher than errors of omission, with a 40% larger area of seagrass suitable habitat predicted in 2004 (23,569 ha, or 236 km^2^) than the observed area of 16,776 Hectare (168 km^2^) [42]. Model coefficients and the threshold value were used to predict seagrass presence for each month. Seagrass suitable habitat area was predicted to be lowest in January 2011, during a major flood, and higher in other months, such as during drought in June 2004 (Fig 5C).

### Effect of sediment loads on seagrass suitable habitat area

**Fig 6:**
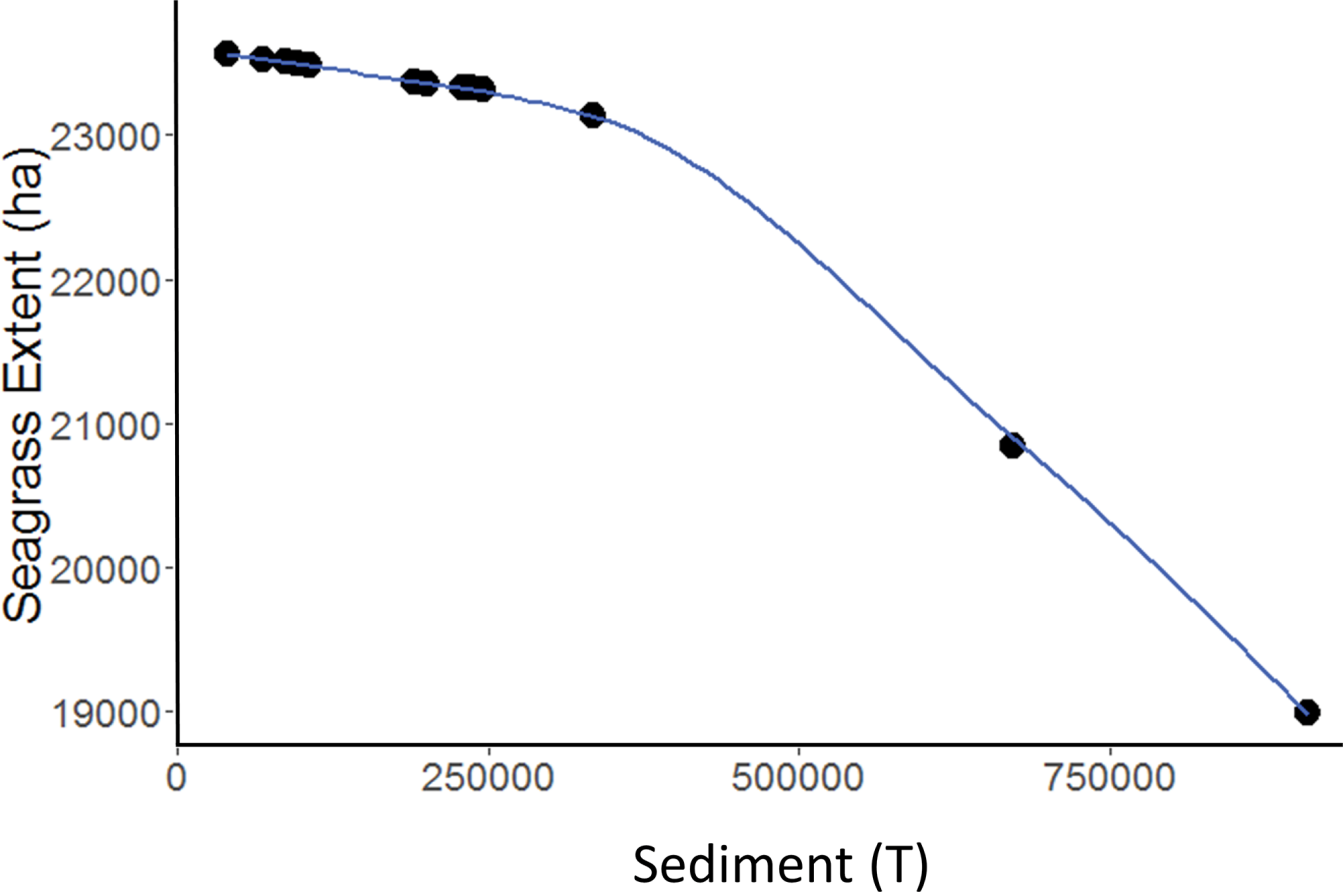
Relationship between sediment loads and seagrass habitat area. Effect of annual sediment load (Tonnes) on area of seagrass suitable habitat (Hectares) in Moreton Bay, Southeast Queensland, Australia. Points represent model estimates for each year from 2001–2013 and the line is a linear model fit of the data.

Increases in sediment loads resulted in non-linear decreases in the area of seagrass suitable habitat (Fig 6). The shape of the relationship between seagrass suitable habitat area in each year and sediment was best fit with a quadratic function (adjusted r^2^ = 0.977, p << 0.001). The model estimated mean seagrass area (*Â*) in a given year in Hectares as:

$$\hat{A}=2.36\times 10^{4}-5.66\times 10^{-9}(S^{2})$$ (6)

where *S* is the total annual (July to June) sediment in Tonnes (Bold text in Table 2) (see [17]).

**Table 2:** Results of statistical analyses of the effect of sediment loads on seagrass extent in Moreton Bay, Australia.

Time-scale	Metric	Results of statistical analysis						
		Thresh	Intercept	Sediment	Sediment^2^	F	DF	p
Annual	sens = spec	0.11	3.95E+04	1.44E-03	-9.46E-09	2115	2,10	<0.01
	**kappa**	**0.22**	**2.36E+04**	**-5.66E-09**	**NA**	**2848**	**2,11**	**<0.01**
	maxPC	0.44	6.43E+03	-1.50E-03	-5.02E-10	1162	2,10	<0.01
	default	0.50	3.80E+03	-8.46E-04	-3.28E-10	1145	2,10	<0.01
Monthly	sens = spec	0.11	3.96E+04	3.63E-03	-1.40E-07	6372	2,159	<0.01
	kappa	0.22	2.36E+04	-3.46E-03	-7.99E-08	3825	2,159	<0.01
	maxPC	0.44	6.41E+03	-1.03E-02	-3.90E-09	4092	2,159	<0.01
	default	0.50	3.80E+03	-5.88E-03	-2.68E-09	3354	2,159	<0.01

Best model fits for models describing the effect of sediment loads from catchments on the extent of habitat suitable for seagrass. Bold text indicates the main result, with other rows indicting results from sensitivity analyses.

### Sensitivity analysis

**Fig 7:**
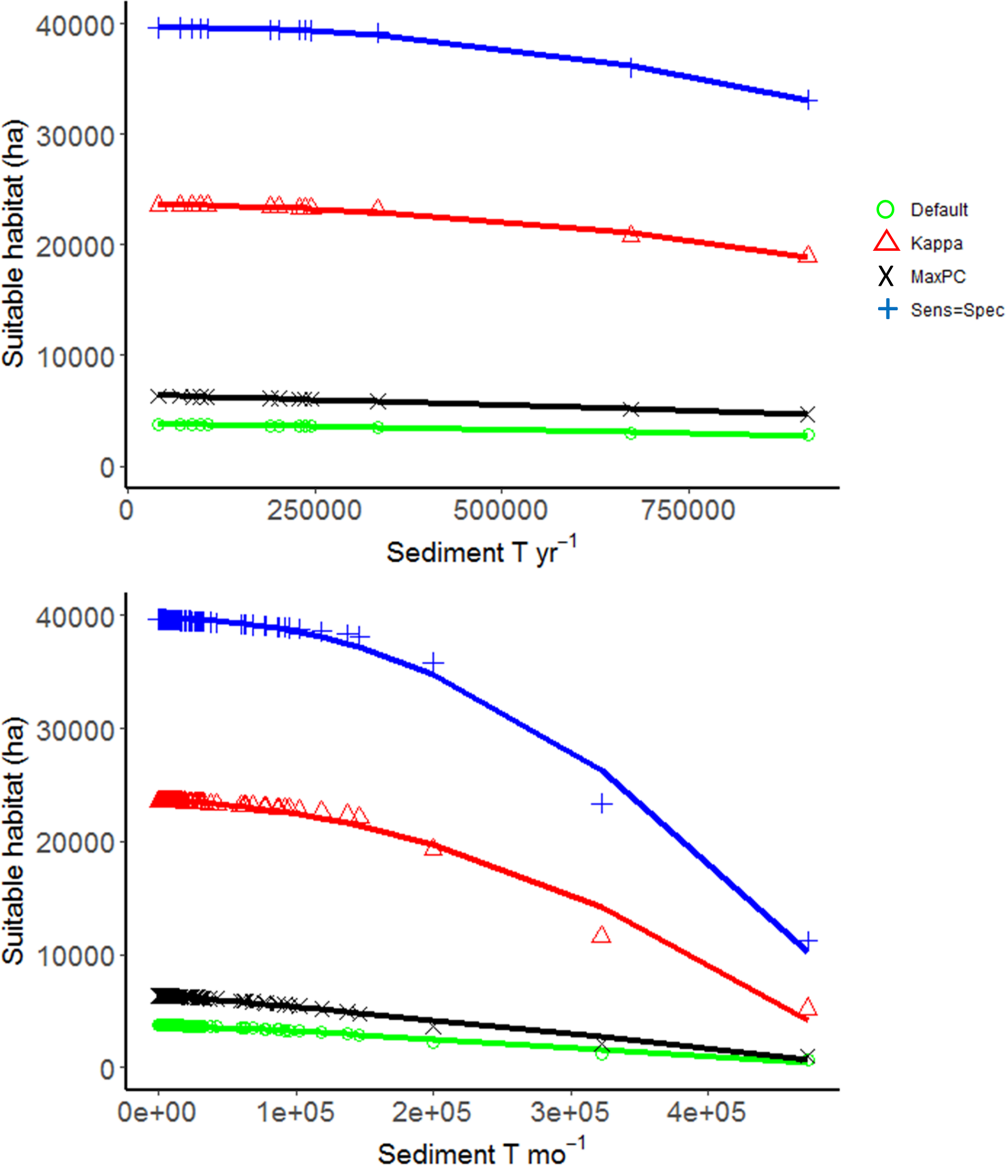
Sensitivity analysis of the effects time-scale and threshold value on the relationship between sediment loads and seagrass habitat area. Effect of sediment load on the extent of seagrass suitable habitat (Ha) in Moreton Bay, Southeast Queensland, Australia. Points represent model estimates for each year from 2001–2013 and lines are linear model fits of the data. a) Data aggregated over annual time-scales; b) Data aggregated over monthly time-scales. Coloured lines within plots indicate results obtained using four different threshold cut-off values. The threshold value is used to assign seagrass presence vs. absence from predicted probability of occurrence based on the habitat distribution model.

The choice of timescale (monthly, yearly) or threshold value to select presence versus absence (optimising for Kappa, Max PCC, Default, or Sensitivity = Specificity) affected the magnitude–but not the overall shape–of the response of seagrass suitable habitat extent to sediment loads (Fig 7, Table 2). In all instances, the relationship between sediment loads and seagrass suitable habitat extent is non-linear, with large increases in sediment load causing disproportionally high decreases in the area of habitat suitable for seagrass (Fig 7). The model formulation was slightly different between the analyses for the main result and the sensitivity analysis. The best model for the sensitivity analyses included both *S* and *S*^*2*^, in contrast to the main model result which included only *S*^*2*^ (Table 2). We suggest the results for annual time-scales are more accurate than those for monthly time-scales, because seagrass are able to adapt or withstand to short term decreases in the light environment [75] and the light environment over annual time-scales is a better predictor of seagrass distribution than the light environment over monthly time-scales [76]. As the threshold value increases, the total area of seagrass suitable habitat decreases for a given sediment load (Fig 7). Over annual, but not necessarily monthly time-scales, the threshold value has a larger effect on the extent of seagrass suitable habitat than sediment load for a particular threshold value (Fig 7).

## Discussion

Objective-based integrated land-sea planning projects require information on the relationship between sediment delivered from catchments to the coast and metrics of the impact of those sediments on the marine environment. The impacts of sediment run-off used in integrated land-sea models have been typically based on linear diffusive models of sediment dispersion (e.g. [1, 14]), and have not factored in the mechanism whereby suspended sediments impact marine species. Here, we provide an advance on this approach by factoring in mechanistic impacts of run-off on benthic light availability–and therefore habitat suitability–for a photosynthetic habitat (seagrass meadows) in Moreton Bay, Australia. Output of the modelling approach provides an important, yet previously missing, linkage between seagrass suitable habitat and terrestrial sediment inputs to coastal waters.

There are four key steps to our approach–data generated from the first two steps linking sediment to water clarity and benthic light availability can be used directly in other applications given data availability. Steps 3 and 4 require information on the relationship between water clarity and ecosystem extent and/or function, which may differ among ecosystems. For instance, the impact of sediment loads on algae, such as kelp, could be modelled in a similar manner to seagrass meadows, because benthic light availability is commonly used as a predictor variable in spatial models of those systems (e.g. [77, 78]). Coral reefs are often measured or modelled in terms of percent cover of coral (e.g. [79, 80]), and steps 3 and 4 in our approach may need to be modified accordingly. For instance, risk scores based on exposure to sediment plumes have been related to % cover of seagrass and corals [25]. Predictions of impact of sediment on coral cover may be improved by factoring in relationships between, and interactions among, coral biodiversity, algal abundance, or reef fish assemblages to water quality [79–82].

The sediment loads used to generate the curve in Fig 6 represent climatic variability among years, where there are higher sediment loads in years with higher rainfall. For this relationship to be applied in integrated land-sea conservation models, it would be assumed that changes in land-use would cause sediment to either increase or decrease, and that downstream water clarity would change similarly [17]. Factoring in impacts of variation in both climate and land-use on water clarity and downstream habitat suitability is an important area of future research. Our results suggest that the relationship between sediment load and the amount of suitable seagrass habitat is non-linear–larger amounts of sediment do disproportionately more damage to seagrass ecosystems (see Fig 6). This super-additive impact of sediment means that substantial improvements in marine ecosystem health might be possible without dramatic changes in sediment inputs, depending on where on the curve the system is currently located.

Environmental disturbances, such as those caused by extreme events or human activities, can have dramatic and non-linear impacts on geomorphic and ecologic systems [80, 83]. Predicting impacts of extreme rainfall events on riverine systems is challenging [83, 84], and field measurements quantifying the impacts of storm events on sediment loading in riverine systems are required. It is difficult to predict the impact of large flood events on marine ecosystems; reduced benthic light availability from pulses in turbidity cause die-offs of sensitive species such as seagrass in some, but not all, instances. For instance, in Hervey Bay, Queensland, 1000 km^2^ of seagrass was lost after two major floods and a cyclone in 1992 [85]. In contrast, significant flooding of the Brisbane River occurred in January 2011 [73], delivering ca. 860,000 T of sediment to the coast, but widespread losses of seagrass were not reported. Benthic light predictions for January 2011 suggested that nearly all of the seagrass habitat in Moreton Bay would be affected (see Fig 5C). While losses of seagrass did occur following flooding [86], by June 2011 no substantial losses of seagrass were recorded bay-wide [41]. This may be because seagrass can adapt to periodic reductions in benthic light intensity [87], or because losses were rapidly compensated for by new colonisation. Our model does not capture those processes explicitly, but instead smooths out variability imposed by episodic events by integrating over annual time scales. We investigated how the choice of timescale affects results in a sensitivity analysis. While the magnitude of the effect of timescale on the response of seagrass suitable habitat extent to sediment loads varies, the overall trend, of non-linear decreases in seagrass suitable habitat extent as sediment loads increase, is not affected by timescale.

This study assessed a land-sea system with a century and a half long legacy of human modification [39], raising questions about historical baselines. For instance, what were the sediment loads prior to European colonisation, how clear was the water, and what area of seagrass habitat could have been supported under those conditions? River sediment loads in Queensland increased between 2- and 14-fold following the arrival and settlement of Europeans in the region [88–90]. In Southeast Queensland, sediment loads from the area below dams are estimated to be 25 to 70 times greater than prior to the beginning of vegetation clearing [66]. Coastal water clarity and the area of seagrass habitat supported prior to European colonisation–and extensive land clearing–is more challenging to estimate.

There are a number of caveats to this research, including limitations to the input data and models. First, uncertainties in the input data used in the modelling approach (Table 1) will influence the model outputs. For instance, the seagrass map used to develop the habitat distribution model was generated using field data and remote sensing imagery with >75% of the region mapped with high reliability [42]. Errors in input data could influence the predictions of sediment run-off, or the distribution of false positives and negatives in the habitat distribution model. Bay-wide seagrass maps exist only for 2004 and 2011, so we cannot verify seagrass habitat area observed in each year [41]. Annual maps from 1972 to 2010 exist for clear water regions [91], but the same approach cannot be used in turbid inshore regions without field data, which cannot be obtained retroactively. Second, there are limitations to the modelling approaches. For instance, the Source model simulates sediment generation in a river catchment, and may not necessarily represent in-stream sediment concentrations or loads. Furthermore, it is principally designed to predict run-off event loads, which may present difficulties in matching output to monthly *in-situ* water quality observations at the coast. We addressed that issue by explicitly quantifying the effect of sediment loads on coastal water clarity in step 2.

Habitat distribution models are a powerful tool for estimating suitable habitat extent, but are imperfect, and factoring in all sources of uncertainty poses a substantial challenge [92]. Habitat distribution models do not factor in biologic factors such as growth, reproduction, or colonisation [93], and as such may overestimate how quickly species can colonise after disturbance or establish in newly suitable areas. The habitat distribution model developed and used in [44–46, 59] and applied in the present study correctly classified seagrass presence vs. absence in 85% of cases, and captures much of the overall spatial distribution of seagrass habitat, but does include errors of omission and commission. False positives may indicate that: (1) the habitat is suitable but that biological factors have prevented the establishment of seagrass; (2) there are areas of omission of seagrass presence from the habitat map used to train the model; or (3) the model could be improved by the inclusion of additional environmental variables. False negatives may indicate use of a threshold value that is too high, or that the environment has changed but the ecological system has not yet responded. Our sensitivity analysis demonstrated that the selection of a threshold value to delineate presence vs. absence from probability of presence, and which influences the incidence and distribution of false positives and false negatives, has a large impact on the extent of habitat predicted to be suitable for seagrass for a given sediment load; however, the threshold value does not affect the overall form of the relationship between sediment load and seagrass extent.

This research could be significantly improved in a number of ways. Using process-based models which fully integrate land-use change, freshwater flows, sediment erosion, coastal oceanography, and seagrass population dynamics would provide a significant improvement over the approach presented here. Such approaches have been developed for particular areas, such as the Great Barrier Reef [94], but are not currently available for most regions. Systematic long-term monitoring of catchment erosion, freshwater and estuarine water quality, and coastal marine ecosystems over appropriate spatial and temporal scales would provide ‘real world’ data that could be used to better understand the underlying ecological process and provide validation to models. For this approach to work, repeatable methods for mapping seagrass over large spatial scales and environmental gradients [95] would need to be implemented over regular time intervals. Fully accounting for and propagating errors in the modelling process would provide a significant advance, but would be complex, and is not currently standard practice in this type of application. Future approaches should consider moving beyond modelling habitat extent, and include, for instance, predictions of percent cover, density, or species composition. Sediment run-off from land threatens the functions and services provided by coastal marine habitats, thus reduction of run-off is urgently required [96, 97]. We recommend that future research aim to: (1) transfer this approach to other systems or locations; (2) devise methodologies to do so in more data limited regions [20, 24]; (3) disentangle the relative contributions of land-use change vs. rainfall on sediment run-off to marine habitat extent; (4) factor in the biological responses of marine organisms to changes in environmental conditions. Ideally, integrated land-sea conservation planning will move towards objective-based approaches, and the methodology presented herein can help inform this process (e.g. [17]).

## Supporting information

S1 Table Total suspended sediment data. Total suspended sediment (TSS) data output from the Source model of Southeast Queensland, Australia. Model output on daily time-step of TSS from the three main rivers (Pine, Lower Brisbane, and Logan/Albert) entering into Moreton Bay, QLD.
(XLSX)
